# Pattern-Recognition Receptor Agonist-Containing Immunopotentiator CVC1302 Boosts High-Affinity Long-Lasting Humoral Immunity

**DOI:** 10.3389/fimmu.2021.697292

**Published:** 2021-11-18

**Authors:** Luping Du, Liting Hou, Xiaoming Yu, Haiwei Cheng, Jin Chen, Qisheng Zheng, Jibo Hou

**Affiliations:** ^1^ Institute of Veterinary Immunology & Engineering, Jiangsu Academy of Agricultural Sciences, Nanjing, China; ^2^ National Research Center of Engineering and Technology for Veterinary Biologicals, Jiangsu Academy of Agricultural Sciences, Nanjing, China; ^3^ Jiangsu Co-innovation Center for Prevention and Control of Important Animal Infectious Diseases and Zoonoses, Yangzhou, China; ^4^ Jiangsu Key Laboratory for Food Quality and Safety-State Key Laboratory Cultivation Base, Ministry of Science and Technology, Nanjing, China

**Keywords:** PRR agonist, NP, long-term humoral immunity, anti-apoptotic transcription factors, CVC1302

## Abstract

Ideally, a vaccine should provide life-long protection following a single administered dose. In our previous study, the immunopotentiator CVC1302, which contains pattern- recognition receptor (PRR) agonists, was demonstrated to prolong the lifetime of the humoral immune response induced by killed foot-and-mouth disease virus (FMDV) vaccine. To elucidate the mechanism by which CVC1302 induces long-term humoral immunity, we used 4-hydroxy-3-nitrophenylacetyl (NP)-OVA as a pattern antigen and administered it to mice along with CVC1302, emulsified together with Marcol 52 mineral oil (NP-CVC1302). From the results of NP-specific antibody levels, we found that CVC1302 could induce not only higher levels of NP-specific antibodies but also high-affinity NP-specific antibody levels. To detect the resulting NP-specific immune cells, samples were taken from the injection sites, draining lymph nodes (LNs), and bone marrow of mice injected with NP-CVC1302. The results of these experiments show that, compared with mice injected with NP alone, those injected with NP-CVC1302 had higher percentages of NP+ antigen-presenting cells (APCs) at the injection sites and draining LNs, higher percentages of follicular helper T cells (TFH), germinal center (GC) B cells, and NP+ plasma-blasts in the draining LNs, as well as higher percentages of NP+ long-lived plasma cells (LLPCs) in the bone marrow. Additionally, we observed that the inclusion of CVC1302 in the immunization prolonged the lifetime of LLPCs in the bone marrow by improving the transcription expression of anti-apoptotic transcription factors such as Mcl-1, Bcl-2, BAFF, BCMA, Bax, and IRF-4. This research provides a blueprint for designing new generations of immunopotentiators.

## Introduction

Activation of PRRs triggers cell signaling, which stimulates the production of proinflammatory cytokines, chemokines, and type I interferons (IFNs) that support the subsequent development of appropriate pathogen-specific adaptive immunity; thus, PRR activation can be harnessed to accelerate and enhance the induction of vaccine- specific responses (1–3).

To date, four major classes of PRRs have been described, including Toll-like receptors (TLRs), nucleotide-binding oligomerization domain (NOD)-like receptors (NLRs), C- type lectin receptors (CLRs) and retinoic acid-inducible gene-1 (RIG-1)-like receptors (4). Nowadays, an increasing focus has been to utilize natural ligands or synthetic agonists for the four classes of PRRs, either alone or with various formulation. CVC1302, which contains three types of PRR agonists, was demonstrated to boost the long-term humoral immunity induced by killed FMDV vaccine (5).

Our previous study found that immunization with killed FMDV vaccine adjuvanted with CVC1302 induces the establishment of an immunocompetent microenvironment at the injection site, which promotes the recruitment of APCs, especially dendritic cells (DCs) (6). However, owing to the lack of available fluorescently labeled FMDV- specific antibodies, we were unable to assess the ability of CVC1302 to promote the levels of antigen-positive APCs infiltrated in the injection sites and draining LNs in that previous study. Here, we circumvented that issue by utilizing NP-PE as a pattern antigen to analyze the efficient antigen-positive APCs which had the potential to deliver the antigen to T or B cells in the draining LNs and assessed if the effect was enhanced by the inclusion of CVC1302 in the vaccine. As previous study showed that long-term humoral immunity induced by CVC1302-adjuvanted serotype O foot-and-mouth disease inactivated vaccine correlates with promoted TFH and thus germinal center responses in mice, and TFH were found to select high-affinity B cells during affinity maturation, so in this study, we utilized NP-OVA as a pattern antigen to analyze the high-affinity NP-specific antibody levels. Sustained humoral immunity following vaccination is mediated mainly by two types of cells: memory B-cells (MBCs) and long-lived plasma cells (LLPCs) (7). Because MBCs do not actively secrete antibody in the absence of antigen-specific stimulation, CVC1302-induced LLPCs were hypothesized to be the cell type responsible for mediating long-term antibody production.

In the present study, we firstly utilized NP-PE as a pattern antigen, adjuvanted with CVC1302, to assess the ability of APCs to capture antigen and subsequently transport it to the draining LNs, thus initiating humoral immune responses. Then we used NP-OVA as pattern antigen to evaluate the ability of CVC1302 to induce high-affinity NP-specific antibody levels. Finally, we assessed the percentages and anti-apoptotic ability of murine NP-specific LLPCs induced by NP-OVA adjuvanted with CVC1302. Our overall goal was to elucidate the mechanisms contributing to long-term humoral immunity that are enhanced by CVC1302; this information will provide new insights toward the development of a new generation of immunopotentiators.

## Material and Methods

### Mice

Six-week-old, female, pathogen-free BALB/c mice were purchased from Yangzhou University (Yangzhou, China). The study and protocol were both approved by the Science and Technology Agency of Jiangsu Province (approval number: NKYVET 2015-0066) and by the Jiangsu Academy of Agricultural Sciences Experimental Animal Ethics Committee. All efforts were made to minimize animal suffering. All animal studies were performed in strict accordance with the guidelines outlined in the Jiangsu Province Animal Regulations (Government Decree No. 45).

### Antigen, Adjuvant, and Immunizations

Killed FMDV serotype O (KV) was a kind gift from China Agricultural Vet. Bio. Science and Technology Co. Ltd. (CAVI, Lanzhou, China). NP-PE and NP-OVA were both purchased from Biosearch Technologies (USA). CVC1302 was prepared in accordance with the procedures outlined in the Chinese patent (registration number: 201310042983.0, **Supplemental Figure 1**). Briefly, CVC1302 is composed of MDP, MPL, and β-glucan, all of which were purchased from InvivoGen (San Diego, CA, USA). All components were dissolved in sterile water at the appropriate concentrations to form the aqueous phase. Marcol 52 mineral oil (ESSO, Paris, France) formed the oil phase. Prior to vaccination, CVC1302 (aqueous phase) was mixed with NP-PE or NP-OVA at a ratio of 1:25, then emulsified with Marcol 52 mineral oil at a ratio of 1:2; both of these were designated NP-CVC1302, whereas NP-PE or NP-OVA mixed directly with Marcol 52 mineral oil at a ratio of 1:2 was designated NP. Alternately, CVC1302 (aqueous phase) was mixed with KV at a ratio of 1:9, then emulsified with Marcol 52 mineral oil at a ratio of 1:2; this was designated KV-CVC1302, whereas KV mixed directly with Marcol 52 mineral oil at a ratio of 1:2 was designated KV.

**Test 1.** Mice were randomly divided into three groups and injected intramuscularly in the quadriceps muscles of each hind leg with 50 µL of antigen-adjuvant mixture (100 µL/mouse). Group 1 mice were immunized with Marcol 52 mineral oil alone as a negative control. Group 2 mice were immunized with KV (KV: 0.3 μg/mouse). Group 3 mice were immunized with KV-CVC1302 (KV: 0.3μg/mouse; MDP: 0.2 mg/mouse; MPL: 10 μg/mouse; β-glucan: 0.2 mg/mouse). Samples were collected to analyze the differentiation of plasma-blasts and LLPCs, the apoptotic levels of LLPCs, and the relative expression levels of anti-apoptotic genes correlating with the lifetime of LLPCs.

**Test 2.** Mice were randomly divided into three groups and injected intramuscularly in the quadriceps muscles of each hind leg with 50 µL of antigen-adjuvant mixture (100 µL/mouse). Group 1 mice were immunized with Marcol 52 mineral oil alone as a negative control. Group 2 mice were immunized with NP (NP: 50 μg/mouse). Group 3 mice were immunized with NP-CVC1302 (NP: 50 μg/mouse; MDP: 0.2 mg/mouse; MPL: 10 μg/mouse; β-glucan: 0.2 mg/mouse). Samples were collected to analyze the antibody titers of high-affinity, NP-specific antibody and the differentiation of NP+ plasma-blasts and NP+ LLPCs.

### Quantification of NP-Specific Antibodies in Serum

Serum samples of individual mice were collected at 14, 28, 56, 90, 120, and 150 days post-immunization (dpi). Ninety-six-well plates were coated overnight at 4°C with NP15-BSA (0.5 µg per well, Biosearch Technologies, USA) to capture diverse-affinity anti-NP antibodies or with NP1-BSA (0.5 µg per well, Biosearch Technologies, USA) to capture high-affinity anti-NP immunoglobulin (Ig)G. The levels of IgG1 and IgG2a isotype diverse-affinity and high-affinity anti-NP antibodies at 56 dpi were also determined. The applied protocols were conducted as previously reported (8, 9). Briefly, NP15-BSA or NP1-BSA-coated ELISA plates were blocked with 2% heat-inactivated FBS in PBS for 1 h at room temperature and incubated 1 hour (h) with serum (dilution at 1:200) from mice immunized with NP-CVC1302 or NP. The plates were washed three times and then incubated with HRP-conjugated monoclonal antibodies [anti-IgG (1:5000), anti-IgG1 (1:5000), anti-IgG2a (1:5000); AbD Serotec, UK] for 45 min at 37°C. After another three washes with PBST, wells were incubated with 3, 3’, 5, 5”- Tetramethylbenzidine (TMB) substrate solution (Biopanda Diagnostics, UK) at 37°C for 15 min, and the reaction was stopped by adding 2 M H2SO4. The OD450 was determined by an ELISA reader (BioTek, USA).

### Preparation of Single-Cell Suspensions From Injection Sites, Draining LNs, and Bone Marrow

Single-cell suspensions were generated from harvested quadriceps muscles as previously reported (10). Each cell suspension was centrifuged at 400 ×*g* for 5 min, resuspended in Dulbecco’s Modified Eagle’s Medium (DMEM; Gibco), filtered through a 70-µm nylon mesh (BD Falcon, USA), and stained with fluorescently labeled antibodies for flow cytometric analysis. Single lymphocytes were harvested from draining LNs as previously reported (6). The lymphocytes were prepared using a Medimachine system with a Medicon (50 µm) disaggregator (BD Biosciences, San Diego, CA, USA) and stained with fluorescently labeled antibodies for flow cytometry.

Bone-marrow cells were harvested as previously reported (11). In brief, bone-marrow single-cell suspensions were prepared from the femurs and tibias of vaccinated mice and treated with red blood cell lysis buffer (Beyotime, Shanghai, China), followed by filtering through a 70-µm nylon mesh (BD Biosciences).

### Flow Cytometric Analysis

For the characterization of NP+ APCs, approximately 2×106 cells per sample were incubated with Fc-blocking reagent in phosphate-buffered saline (PBS) containing 1% fetal bovine serum (FBS) for 10 min at 4°C. The cells were then incubated with the following fluorescently labeled antibodies: PE-Cy7-conjugated anti-CD11b, fluorescein isothiocyanate (FITC)-conjugated anti-CD11c, and allophycocyanin (APC)-conjugated anti-Ly6c (BD Biosciences, USA) or PE-Cy7-conjugated anti-CD11b, FITC- conjugated anti-CD11c, and APC-conjugated anti-F4/80 (BD Biosciences, USA). 2×105 cells were acquired by BD Accuri C6, cells were first identified as singlets *via* standard FSC and SSC gating, NP+ cells were gated as NP-PE. The NP+ cDCs were identified as NP+CD11c+CD11b+, NP+ Mo was identified as NP+ CD11c-CD11b+Ly6c+, NP+ Mph was identified as NP+CD11c-CD11b+F4/80+.

For the characterization of TFH cells, approximately 2×10^6^ cells per sample were incubated with Fc-blocking reagent in PBS containing 1% FBS for 10 min at 4°C. The cells were then incubated with the following fluorescently labeled antibodies: PE-Vio770-conjugated anti-CD4, FITC-conjugated anti-CXCR5, PE-conjugated anti-PD-1 (BD Biosciences, USA). 10,000 cells were acquired by BD Accuri C6, cells were first identified as singlets *via* standard FSC and SSC gating, T cells were gated as CD4, TFH cells were then identified as CD4+CXCR5+PD-1+.

For the characterization of NP+ GC B cells, approximately 2×106 cells per sample were incubated with Fc-blocking reagent in PBS containing 1% FBS for 10 min at 4°C. The cells were then incubated with the following fluorescently labeled antibodies: FITC- conjugated anti-B220, PE-Vio770-conjugated anti-GL-7 (BD Biosciences, USA), and NP-PE. For NP labeling, cells were stained with a commercially available kit (eBioscience, USA). 10,000 cells were acquired by BD Accuri C6, cells were first identified as singlets *via* standard FSC and SSC gating, NP+cells were gated as NP-PE. The NP+ GC B cells were identified as NP+B220+GL-7+.For the characterization of NP+ plasma-blasts, approximately 2×106 cells per sample were incubated with Fc-blocking reagent in PBS containing 1% FBS for 10 min at 4°C. The cells were then incubated with the following fluorescently labeled antibodies: FITC-conjugated anti-B220, APC-conjugated anti-CD38 (BD Biosciences, USA), and NP-PE. For NP labeling, cells were stained with a commercially available kit (eBioscience, USA). 10,000 cells were acquired by BD Accuri C6, cells were first identified as singlets *via* standard FSC and SSC gating, NP+ cells were gated as NP-PE. The NP+ plasma-blasts were identified as NP+B220+CD38+.

For the characterization of NP+ LLPCs, approximately 2×106 cells per sample were incubated with Fc-blocking reagent in PBS containing 1% FBS for 10 min at 4°C. The cells were then incubated with the following fluorescently labeled antibodies: FITC-conjugated anti-B220, APC-conjugated anti-CD138 (BD Biosciences, USA), and NP-PE. For NP labeling, cells were stained with a commercially available kit (eBioscience, USA). 10,000 cells were acquired by BD Accuri C6, cells were first identified as singlets *via* standard FSC and SSC gating, NP+ cells were gated as NP-PE. The NP+ LLPCs were identified as NP+B220-CD138+.

Cells were analyzed with a BD Accuri C6 instrument (BD Biosciences, USA). Data analyses were performed using FlowJo version 7.6.1 software. All of the flow cytometry dot plots were shown in **Supplement Figure 2**.

### Anti-Apoptotic Analysis

Cell sorting was performed using a FACS Aria cell sorter (BD Biosciences). LLPCs were enriched (at 90 dpi) by the sorting of cells stained with APC-conjugated anti-B220 and PE-conjugated anti-CD138. Apoptosis was assessed using annexin V-FITC and 7- aminoactinomycin (7-AAD) (BD Biosciences, USA) as described previously with minor modifications (12). Briefly, sorted LLPCs were washed twice with PBS, resuspended in 100 µL of 1× binding buffer, and incubated with 5 µL of annexin V- FITC and 5 µL of 7-AAD at 25°C in the dark for 15 min. The volume was increased to 500 µL for flow cytometry by adding binding buffer. Cells were analyzed using a BD Accuri C6 instrument (BD Biosciences). Data were analyzed using FlowJo version 7.6.1 software.

### Confocal Microscopy

Bone-marrow cells collected at 90 dpi (2×105 cells/well) were placed in a 96-well plate. The morphology of apoptotic LLPC nuclei was assessed by staining the cells with PE- conjugated anti-CD138 and the DNA-binding fluorochrome 4’,6-diamidino-2- phenylindole (DAPI) (Beyotime Biotechnology, China). Samples were visualized at room temperature using a Zeiss LSM700 confocal microscope (Zeiss, Oberkochen, Germany), and images were acquired using Zeiss LSM image browser software (Zeiss).

### Determination of mRNA Levels by Real-Time Quantitative PCR

Real-time quantitative PCR (RT-qPCR) using SYBR green was performed to assess the abundance of anti-apoptotic and pro-apoptotic regulator mRNAs in bone-marrow cells. Total bone-marrow cell RNA was extracted using Trizol (Invitrogen, Carlsbad, CA,USA) and reverse transcribed in a 20-µl reaction mixture. The resulting cDNA product (2 µL) was amplified in a 20-µl RT-qPCR reaction. The primer sequences for all genes, including β-actin (a reference gene), are listed in **Supplemental Table 1**.

### Statistical Analysis

Statistical analysis was performed using GraphPad Prism version 5 (GraphPad Software, San Diego, CA, USA). Differences among groups were assessed using a one- way analysis of variance followed by Tukey’s post-hoc *t*-test. Differences between groups were assessed using a Student’s *t*-test. Values of *p* < 0.05 were considered statistically significant. All data shown in the manuscript are expressed as means ± standard errors of the means (SEMs).

## Results

### CVC1302 Enhanced the Ability of APCs to Engulf NP-PE

**Figure 1:**
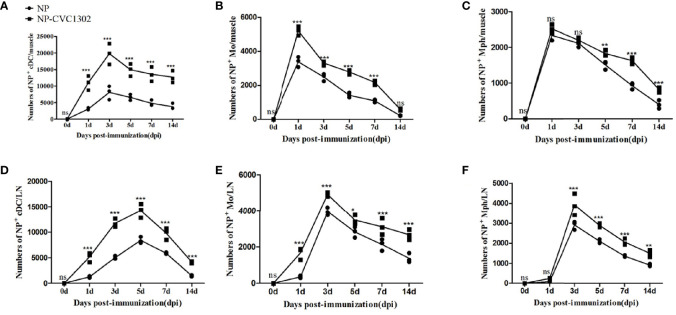
The influx of NP+ APCs at injection sites and translocation of NP+ APCs to draining LNs. Samples were collected at 1, 3, 5, 7, and 14 dpi from mice treated with the immunization protocol described in the Materials and Methods. **(A)** The numbers of NP+ DCs at injection sites. **(B)** The numbers of NP+ Mo at injection sites. **(C)** The numbers of NP+ Mph at injection sites. **(D)** The numbers of NP+ DCs in draining LNs. **(E)** The numbers of NP+ Mo in draining LNs. **(F)** The numbers of NP+ Mph in draining LNs. Values show the mean ± SEMs of 3 muscles from 3 mice per treatment group. *P < 0.05, **P < 0.01, ***P < 0.001, ns, not significant.

As local events at injection sites represent critical steps towards mounting a robust immune response, we investigated the ability of CVC1302 to induce an immunocompetent microenvironment in our previous study (6). However, owing to the lack of an available FMDV-specific antibody, we had limited capacity to analyze the ability of NP+ APCs influx at the injection sites and translocation to the draining LNs in that work. Here, by using NP-PE as the pattern antigen, we were able to detect the NP+ APCs and assess the capacity of CVC1302 to improve the influx of NP+ APCs at the injection sites and translocate to draining LNs, where it can activate T and B cells. The numbers of NP+ APCs at the injection sites or draining LNs were measured by using flow cytometry. The results indicate that significantly higher numbers of NP+ DCs (NP+, CD11c+, CD11b+ cells) were observed at the injection sites of mice immunized with NP- CVC1302 as compared with those of mice immunized with NP alone, most notably at 3 dpi, which is consistent with the findings from our previous study. At the injection sites, peak numbers of NP+ DCs were observed at 3 dpi for both the NP-CVC1302 and NP groups, and peak numbers of NP+ Mph (NP+, CD11b+, CD11c−, F4/80high cells) and NP+ Mo (NP+, CD11b+, CD11c−, Ly6C+ cells) were observed at 1 dpi (**Figure 1A**). The numbers of NP+ APCs in the draining inguinal LNs were concurrently analyzed using flow cytometry. In the inguinal LNs, the highest number of NP+ DCs was detected at 5 dpi for both the NP-CVC1302- and NP-immunized groups, and the highest number of NP+ Mph and NP+ Mo were detected at 3 dpi (**Figure 1B**). Both at the injection sites and in the inguinal LNs, NP+ DCs formed the highest percentage of cells among all NP+ APCs in both the NP-CVC1302 and NP groups.

### CVC1302 Induces High-Affinity Ig Responses

**Figure 2:**
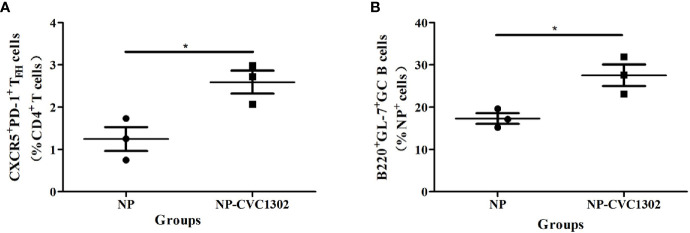
NP^+^GC B cells and TFH percentages in immunized mice. **(A)** Percentages of NP^+^ GC B cells and **(B)** TFH in mice immunized once with Marcol 52, NP, or NP-CVC1302. Samples were collected at 14 dpi. Values show the mean ± SEMs of 3 draining LNs from 3 mice per treatment group. *P < 0.05.

It was previously demonstrated that CVC1302 promotes the differentiation of TFH cells (13), which was also demonstrated as shown in **Figure 2**. Because TFH cells regulate germinal center (GC) size, restrict low-affinity B-cell entry into the GC, support high- affinity B-cell occupancy of the GC, and select high-affinity B cells during affinity maturation (14), the titers of high-affinity NP-specific antibody were measured after using flow cytometry.

The results indicate that significantly higher numbers of NP+ DCs (NP+, CD11c+, CD11b+ cells) were observed at the injection sites of mice immunized with NP- CVC1302 as compared with those of mice immunized with NP alone, most notably at 3 dpi, which is consistent with the findings from our previous study. At the injection sites, peak numbers of NP+ DCs were observed at 3 dpi for both the NP-CVC1302 and NP groups, and peak numbers of NP+ Mph (NP+, CD11b+, CD11c−, F4/80high cells) and NP+ Mo (NP+, CD11b+, CD11c−, Ly6C+ cells) were observed at 1 dpi (**Figures 1A–C**). The numbers of NP+ APCs in the draining inguinal LNs were concurrently analyzed using flow cytometry. In the inguinal LNs, the highest number of NP+ DCs was detected at 5 dpi for both the NP-CVC1302- and NP-immunized groups, and the highest number of NP+ Mph and NP+ Mo were detected at 3 dpi (**Figures 1D–F**). Both at the injection sites and in the inguinal LNs, NP+ DCs formed the highest percentage of cells among all NP+APCs in both the NP-CVC1302 and NP groups.

### CVC1302 Induces High-Affinity Ig Responses

**Figure 3:**
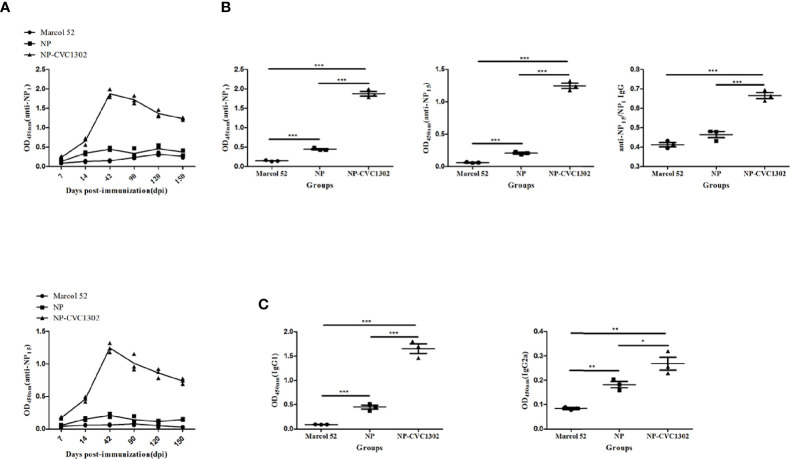
Effect of CVC1302 on affinity maturation. **(A)** Kinetics of diverse-affinity anti- NP IgG (anti-NP15, *upper*) and high-affinity anti-NP IgG (anti-NP1, *lower*) antibody levels following immunization with Marcol 52, NP, or NP-CVC1302. **(B)** Levels of diverse-affinity anti-NP IgG (*left*), high-affinity anti-NP IgG (*center*), and affinity maturation of anti-NP IgG (ratio of anti-NP1 to anti-NP15 IgG, *right*) as measured by ELISA at 42 dpi. **(C)** Total anti-NP IgG1 (left) and IgG2a (right) levels were measured by ELISA at 42 dpi as in **(A)**. Values show the mean ± SEMs of 7 mice per treatment group. *P < 0.05, **P < 0.01, ***P < 0.001.

It was previously demonstrated that CVC1302 promotes the differentiation of TFH cells (13), which was also demonstrated as shown in **Figure 2**. Because TFH cells regulate germinal center (GC) size, restrict low-affinity B-cell entry into the GC, support high- affinity B-cell occupancy of the GC, and select high-affinity B cells during affinity maturation (14), the titers of high-affinity NP-specific antibody were measured after immunization with NP-CVC1302. Mice immunized with NP-CVC1302 displayed higher titers of both total and high-affinity anti-NP IgG, as well as of IgG1 and IgG2a, as compared with mice immunized with NP (**Figure 3**). As shown in **Figure 3C**, CVC1302 had a strong effect on the production of IgG1, but a minimal effect on the production of IgG2a. These conclusions are supported by the results of a statistical analysis (**Supplemental Table 2**).

### CVC1302 Increases LLPC Production in the Bone Marrow

In our previous study, we found that KV-CVC1302 could induce long-term humoral immunity (up to 5 months) in mice and pigs (5, 6). Because the mice and pigs in that study received only one dose of KV-CVC1302, LLPCs were considered to be the most likely mediators of the observed sustained antibody production (15). To test this hypothesis, we analyzed plasma-blasts in the inguinal LNs and LLPCs in the bone marrow of KV-CVC1302-immunized mice, as well as NP+ plasma-blasts and NP+ LLPCs in NP-CVC1302-immunized mice, by using flow cytometry.

**Figure 4:**
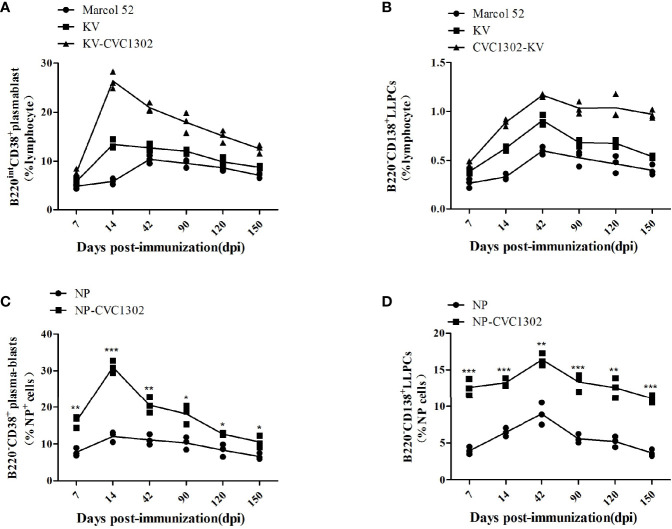
Plasma-blast and LLPC percentages in immunized mice. **(A, B)** Percentages of plasma-blasts **(A)** and LLPCs **(B)** in mice immunized once with Marcol 52, KV, or KV- CVC1302. **(C, D)** Percentages of NP+ plasma-blasts **(C)** and NP+ LLPCs **(D)** in mice immunized once with Marcol 52, NP, or NP-CVC1302. Samples were collected at 7, 14, 42, 90, 120, and 150 dpi. Values show the mean ± SEMs of 3 draining LNs from 3 mice per treatment group. *P < 0.05, **P < 0.01, ***P < 0.001.

As shown in **Figures 4A, B**, both plasma-blasts and LLPCs in mice immunized with KV-CVC1302 or KV alone reached their peak levels at 14 and 42 dpi, respectively. At each sampling point, the percentages of plasma-blasts and LLPCs in KV-CVC1302- immunized mice were higher than those in KV-immunized mice. All differences between the two groups, except that at 7 dpi, were statistically significant. Because bulk plasma-blasts and LLPCs each include both FMDV-specific and nonspecific cells, the differences between the two groups are more informative compared with the absolute numbers of cells. These conclusions are supported by the results of a statistical analysis (**Supplementary Table 3**). The absolute percentages of NP+ plasma-blasts and NP+ LLPCs were also measured to assess the ability of CVC1302 to enhance humoral immune responses. Similar to the results for plasma-blasts and LLPCs induced by KV- CVC1302 or KV, the highest percentages of NP+ plasma-blasts or NP+ LLPCs were observed at 14 or 42 dpi, respectively, and the largest difference in NP+ LLPCs between the groups of mice immunized with NP-CVC1302 and NP were also observed at 42 dpi (**Figures 4C, D**).

### CVC1302 Alleviates LLPC Apoptosis

**Figure 5:**
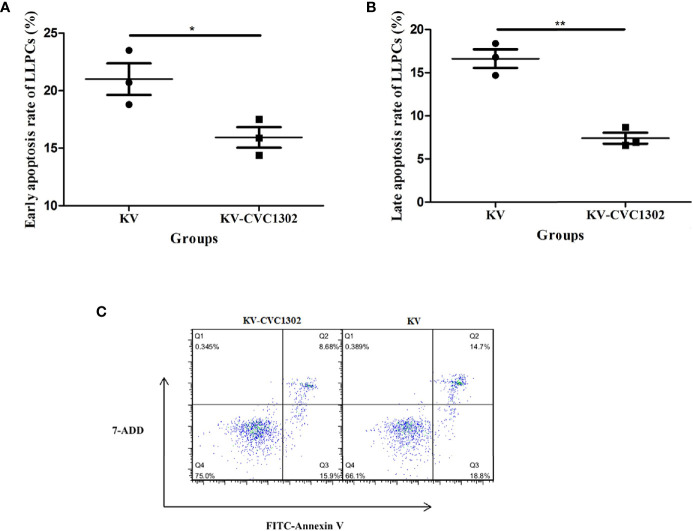
Percentages of early and late apoptosis in LLPs from immunized mice. The percentages of early apoptosis **(A)** and late apoptosis **(B)** in LLPCs from mice immunized with KV or KV-CVC1302. **(C)** The flow cytometry gating strategies of the apoptosis of LLPCs. Samples were collected at 90 dpi. Data are presented as the mean ± SEM. *P < 0.05, **P < 0.01.

As shown in **Figure 4B**, there were declines in the percentages of LLPCs in KV- and KV- CVC1302-immunized mice at 90 dpi. However, the degree of LLPC decline was non-uniform. We speculated that this difference might arise from differential LLPC apoptosis. To test this hypothesis, the population of LLPCs was enriched and stained with annexin V-FITC/7-AAD. As shown in **Figures 5A–C**, the rates of early and late apoptosis of LLPCs from KV-CVC1302-immunized mice were significantly lower than those of KV-immunized mice. Thus, CVC1302 may prolong the survival of LLPCs that are induced by a killed FMDV vaccine. The different degrees of LLPC apoptosis between KV- and KV-CVC1302-immunized mice could explain the differences in bone-marrow LLPC percentages between the two groups.

**Figure 6:**
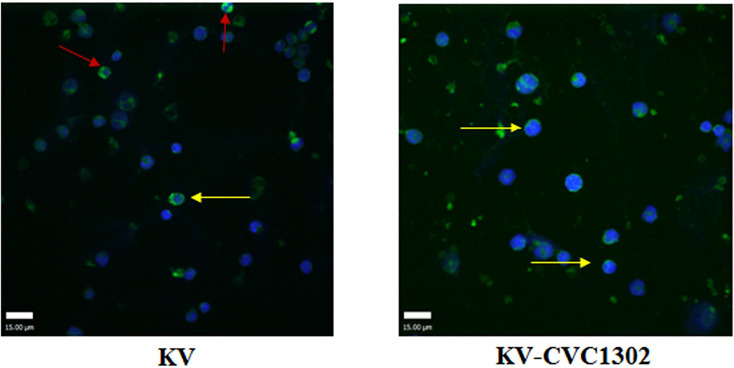
The apoptotic nuclei of LLPCs from mice immunized with KV (left) or KV- CVC1302 (right). Samples were collected at 90 dpi. LLPCs are shown in green, and the nuclei (DAPI) are shown in blue. The red and yellow arrows indicate shrunken and normal LLPC nuclei, respectively.

To visually observe LLPC apoptosis, LLPC nuclei were examined *via* confocal microscopy. As shown in **Figure 6**, apoptotic nuclei exhibiting compaction and pleomorphism were observed in CD138+ LLPCs from KV-immunized mice.

### CVC1302 Inhibits LLPC Apoptosis by Influencing Apoptotic Regulators

**Figure 7:**
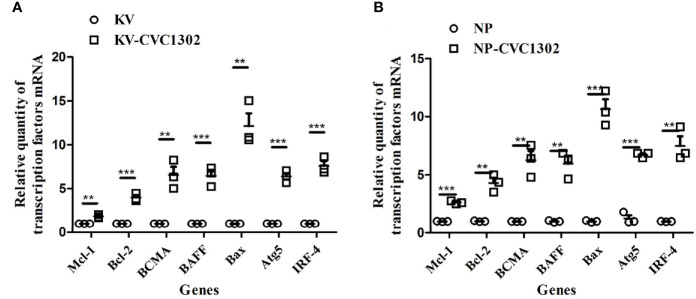
Effect of CVC1302 adjuvantation on the expression of anti-apoptotic regulators essential for extending the life-time of LLPCs. Bone-marrow cells were isolated at 90 dpi, and RNA was extracted from these cells by using Trizol. The expression levels of anti-apoptotic regulators [**(A)** represents test 1, **(B)** represents test 2] were detected by real- time RT-PCR. Results are expressed as the mean ± SEM. **P < 0.01, ***P < 0.001.

The lifespan of LLPCs depends on both cell-intrinsic programs and extrinsic factors (16). We assessed whether CVC1302 improved the longevity of LLPCs through intrinsic factors by applying qRT-PCR. As shown in **Figure 7A**, compared with KV- immunized mice, regulators of plasma cell longevity including cell survival signals (Bcl-2, Mcl-1, IRF4, BCMA, BAFF) (17–21) were expressed at significantly higher levels in KV-CVC1302-immunized mice. The relative transcription levels of anti- apoptosis genes were also measured in mice immunized with NP-CVC1302 or NP. Similar to the results shown in **Figure 7B**, the anti-apoptosis genes in NP-CVC1302- immunized mice had higher transcription levels as compared with those in NP- immunized mice. Previous studies have shown that autophagy is required for plasma- cell homeostasis and long-lived humoral immunity (22), so we also compared the expression levels of Atg5 between LLPCs from KV-immunized and KV-CVC1302- immunized mice. As shown in **Figure 7**, CVC1302-adjuvanted KV or NP could induce a higher Atg5 transcription level compared with KV or NP alone, respectively.

## Discussion

The most important characteristic of a successful vaccine is the induction of long-term immune responses against pathogens (23). Commercially available killed FMDV vaccines usually induce short-term protective immunity and require the periodic administration of booster doses (24). In our previous study, we showed that a single dose of CVC1302 co-administered with killed serotype O FMDV vaccines could induce long-term humoral immunity in mice and pigs. As reported, at least three injections of FMDV vaccines are needed to pigs from birth to euthanization, what’s more, sows need more vaccinations than fattening pigs to ensure FMD antibody titers for the piglets (5). Therefore, the utility of CVC1302 to FMDV vaccines, not only provide animal welfare, but also reduce the cost. To provide new insights into the development of killed FMDV vaccines, we explored the mechanism of action by which CVC1302 acts in prolonging the humoral immune responses induced by killed FMDV vaccines. Because of the lack of available fluorescently labeled FMDV-specific antibody, we used NP as a pattern antigen for these experiments.

In our study, we found that CVC1302 induced higher percentages of TFH. Due to the characterizations of TFH in GC responses, we detected the total- and high-affinity of NP- specific antibody titers. Consistent with the expected results, CVC1302 could enhance the affinity of antigen-specific antibodies.

LLPCs and MBCs mediate long-lived humoral immunity. Because mice and pigs were vaccinated with KV-CVC1302 only once in our previous study, we hypothesized that the induced long-term antibody responses were mediated mainly by LLPCs. During the activation of systemic immune responses, plasma-blasts are generated in GCs in the secondary lymphoid organs following interactions with TFH cells, after which they migrate to the bone marrow (25). The plasma-blasts can then migrate through the bloodstream to find a survival niche (predominantly located in the bone marrow) (26). These cells secrete high-affinity antibodies that are responsible for the maintenance of long-term antibody titers (7). A flow cytometry analysis of the percentages of plasma- blasts in LNs and of LLPCs in the bone marrow revealed that CVC1302 contributes to higher levels of LLPCs and a longer duration of antibody responses induced by immunization with killed FMDV vaccines. The higher percentage of bone marrow LLPCs in KV-CVC1302-immunized mice than in KV-immunized mice depended on both the differentiation of plasma-blasts in LNs and the expression of anti-apoptotic regulators to prolong the lifetime of antigen-specific LLPCs.

In our previous study, we found that CVC1302 established an immunocompetent microenvironment at the injection sites, then recruited efficient APCs and activated DCs; furthermore, we determined that CVC1302 improved the GC responses induced by FMDV through the promotion of TFH cells. However, because of the lack of an available fluorescently labeled FMDV-specific antibody, the present work used the pattern antigen NP to assess the CVC1302-induced influx of NP+ APCs into injection sites and the differentiation levels of NP+ LLPCs. We further demonstrated that CVC1302 also increased the expression of anti-apoptotic regulators to extend the lifetime of KV-induced LLPCs.

In our previous study, we found that not only individual adjuvants but also dual combinations could not induce humoral immune responses against FMDV as high as CVC1302 when adjuvanted with FMDV inactivated vaccine, as well as the longevity of humoral immunity. In China, the current commercial FMDV vaccine adjuvant was mainly Montanide ISA-206, as in our previous study, we found that the addition of CVC1302 in ISA-206 adjuvanted killed FMDV could further improve the levels and longevity of humoral immune response against FMDV. Several kinds of vaccine adjuvants have been studied for their potency to promote immune response to FMDV vaccines, such as ISCOMs, TLR ligands (poly(I:C; CpG; R-837; R-848 and flagellin) and cytokines (27). However, the study of them adjuvanted with FMDV inactivated vaccine limited to the mice models. In our study, we did not confined ourselves to single kind of TLR ligands, we combined three kinds of TLR ligands to improve the efficiency of FMDV inactivated vaccine. Furthermore, CVC1302 could be used not only as aqueous phase added into FMDV inactivated vaccine at the preparation period but also as oil phase added into FMDV inactivated vaccine before vaccination.

In this study, we found that CVC1302 induces higher levels of high-affinity antibodies and long-term humoral immunity by mediating the longevity of LLPCs. The findings will provide a blueprint for designing new generations of immunopotentiators.

## Data Availability Statement

The original contributions presented in the study are included in the article/**Supplementary Material**. Further inquiries can be directed to the corresponding author.

## Ethics Statement

The study and protocol were both approved by the Science and Technology Agency of Jiangsu Province (approval number: NKYVET 2015-0066) and by the Jiangsu Academy of Agricultural Sciences Experimental Animal Ethics Committee.

## Author Contributions

LD, QS, JC, and JH designed the experiment. Sampling of serum, LNs and bone marrow were mainly performed by LD, LT, and XY. LD and HC analyzed the results with guidance from QS and JH and wrote the main manuscript text. All authors took part in discussion and interpretation of results. All authors read, advised, and approved the final manuscript.

## Funding

This work was supported by the National Natural Sciences Foundation of China (31802220), Jiangsu Agricultural Science and Technology Innovation Fund (CX (20)3096).

## Conflict of Interest

The authors declare that the research was conducted in the absence of any commercial or financial relationships that could be construed as a potential conflict of interest.

## Publisher’s Note

All claims expressed in this article are solely those of the authors and do not necessarily represent those of their affiliated organizations, or those of the publisher, the editors and the reviewers. Any product that may be evaluated in this article, or claim that may be made by its manufacturer, is not guaranteed or endorsed by the publisher.
