# Supplementary material for: Pattern-Recognition Receptor Agonist-Containing Immunopotentiator CVC1302 Boosts High-Affinity Long-Lasting Humoral Immunity
Source: Front Immunol. 2021 Nov 18;12:697292. doi: 10.3389/fimmu.2021.697292 (PMC8637734; doi:10.3389/fimmu.2021.697292)
Supplement: Supplementary file 1 [file DataSheet_1.pdf]

Pattern-recognition receptor agonist-containing immunopotentiator CVC1302 boosts long-lasting humoral immunity

Luping Du[a,b,c,d], Liting Hou[a,b,c,d], Xiaoming Yu[a,b,c,d], Haiwei Cheng[a,b,c,d], Jin Chen[a,b,c,d,*], Qisheng Zheng[a,b,c,d,*], Jibo Hou[a,b,c,d]

a. Institute of Veterinary Immunology & Engineering, Jiangsu Academy of Agricultural Sciences, Nanjing, Jiangsu, China, 210014

b. National Research Center of Engineering and Technology for Veterinary Biologicals, Jiangsu Academy of Agricultural Sciences, Nanjing, Jiangsu, China,210014

c. Jiangsu Co-innovation Center for Prevention and Control of Important Animal Infectious Diseases and Zoonoses, Yangzhou, 225009

d. Jiangsu Key Laboratory for Food Quality and Safety-State Key Laboratory Cultivation Base, Ministry of Science and Technology Nanjing, Jiangsu, China, 210014

*Corresponding authors: E-mails: immun_tech@163.com (Qisheng Zheng); chenjin_abc@163.com (Jin Chen)

Supplemental Table 1

Name	Forward	Reverse
Mcl-1	GCTCCGGAAACTGGACATTA	CCCAGTTTGTTACGCCATCT
Bcl-2	GAAACCCCTAGTGCCATCAA	GGGACGTCAGGTCACTGAAT
BCMA	ATCTTCTTGGGGCTGACCTT	CTTTGAGGCTGGTCCTTCAG
BAFF	CCCCAGACACTTCAGAAGGA	AGGTAGGAGCTGAGGCATGA
Bax	TGAAGACAGGGGCCTTTTTG	AATTCGCCGGAGACACTCG
Atg5	TGTGCTTCGAGATGTGTGGTT	GTCAAATAGCTGACTCTTGGCAA
IRF4	TCCGACAGTGGTTGATCGAC	CCTCACGATTGTAGTCCTGCTT
β-actin	CACTGCCGCATCCTCTTCCTCCC	CAATAGTGATGACCTGGCCGT

Supplemental Table 2

Statistical analysis of the titers of both-affinity (anti-NP_{15}) NP-specific antibody among the groups immunized with Marcol 52, NP or NP-CVC1302. *P<0.05, ** P<0.01, *** P<0.001.

Groups / dpi	Marcol 52 vs NP	Marcol 52 vs NP-CVC1302	NP vs NP-CVC1302
7	*	**	*
14	***	***	**
42	***	***	***
90	ns	***	***
120	ns	***	***
150	ns	***	***

Statistical analysis of the titers of both-affinity (anti-NP_1) NP-specific antibody among the groups immunized with Marcol 52, NP or NP-CVC1302. *P<0.05, ** P<0.01, *** P<0.001.

Groups / dpi	Marcol 52 vs KV	Marcol 52 vs KV-CVC1302	KV vs KV-CVC1302
7	ns	**	ns
14	***	***	***
42	*	***	***
90	*	***	***
120	ns	***	***
150	ns	***	***

Supplemental Table 3

Statistical analysis of the percentages of plasma-blasts in draining lymph nodes among the groups immunized with Marcol 52, KV or KV-CVC1302. *P<0.05, ** P<0.01, *** P<0.001.

Groups / dpi	Marcol 52 vs NP	Marcol 52 vs NP-CVC1302	NP vs NP-CVC1302
7	ns	***	***
14	***	***	**
42	***	***	***
90	ns	***	***
120	*	***	***
150	***	***	***

Statistical analysis of the percentages of LLPCs in bone marrow among the groups immunized with Marcol 52, KV or KV-CVC1302. *P<0.05, ** P<0.01, *** P<0.001.

Groups / dpi	Marcol 52 vs KV	Marcol 52 vs KV-CVC1302	KV vs KV-CVC1302
7	ns	***	ns
14	***	***	***
42	***	***	***
90	**	***	***
120	***	***	***
150	*	***	***

Supplemental Fig. 1

The Chinese patent 201310042983.0, which describe the protocol to develop the combination adjuvants including the concentration of each component and the procedure when mixed with vaccine or antigen.

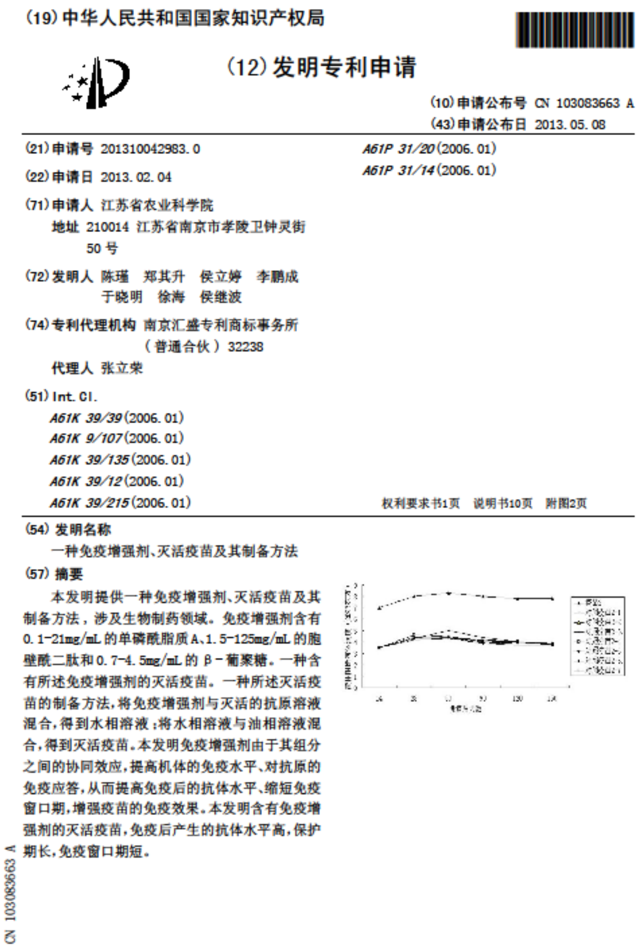
(19) 中华人民共和国国家知识产权局

(12) 发明专利申请

(10) 申请公布号 CN 103083663 A
(43) 申请公布日 2013. 05. 08

(21) 申请号 201310042983. 0
(22) 申请日 2013. 02. 04
(71) 申请人 江苏省农业科学院
地址 210014 江苏省南京市孝陵卫钟灵街 50 号
(72) 发明人 陈瑾 郑其升 侯立婷 李鹏成 于晓明 徐海 侯继波
(74) 专利代理机构 南京汇盛专利商标事务所 (普通合伙) 32238
代理人 张立荣
(51) Int. Cl.
A61K 39/39 (2006. 01)
A61K 9/107 (2006. 01)
A61K 39/135 (2006. 01)
A61K 39/12 (2006. 01)
A61K 39/215 (2006. 01)
A61P 31/20 (2006. 01)
A61P 31/14 (2006. 01)

权利要求书1页 说明书10页 附图2页

(54) 发明名称
一种免疫增强剂、灭活疫苗及其制备方法

(57) 摘要
本发明提供一种免疫增强剂、灭活疫苗及其制备方法，涉及生物制药领域。免疫增强剂含有 0. 1-21mg/mL 的单磷酰脂质 A、1. 5-125mg/mL 的胞壁酰二肽和 0. 7-4. 5mg/mL 的 β－葡聚糖。一种含有所述免疫增强剂的灭活疫苗。一种所述灭活疫苗的制备方法，将免疫增强剂与灭活的抗原溶液混合，得到水相溶液；将水相溶液与油相溶液混合，得到灭活疫苗。本发明免疫增强剂由于其组分之间的协同效应，提高机体的免疫水平、对抗原的免疫应答，从而提高免疫后的抗体水平、缩短免疫窗口期，增强疫苗的免疫效果。本发明含有免疫增强剂的灭活疫苗，免疫后产生的抗体水平高，保护期长，免疫窗口期短。

CN 103083663 A

Supplement Fig. 2.

The flow cytometry gating strategies of all flow cytometry analysis. Fig. 2A The gating strategy for NP$^+$ cDCs and NP$^+$ Mo, Fig. 2B The gating strategy for NP$^+$ Mph. Fig. 2C The gating strategy for T_{FH} cells. Fig. 2D The gating strategy for NP$^+$ GC B

cells. Fig. 2E The gating strategy for NP$^+$ plasma-blasts. Fig. 2F The gating strategy for NP$^+$ LLPCs.

Fig.2A

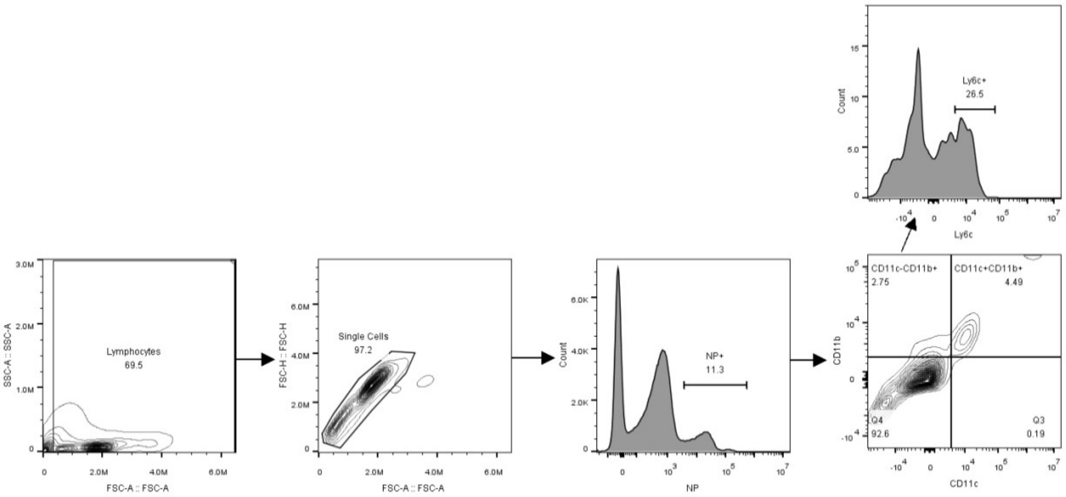

Fig. 2B

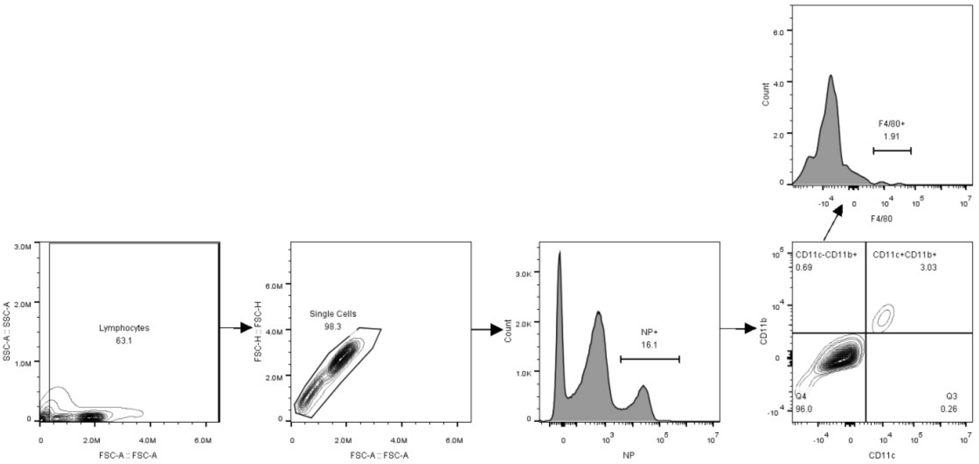

Fig. 2C

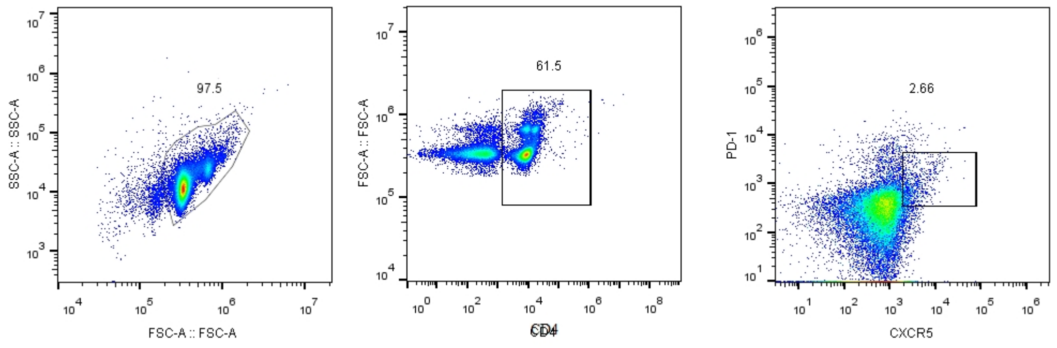

Fig. 2D

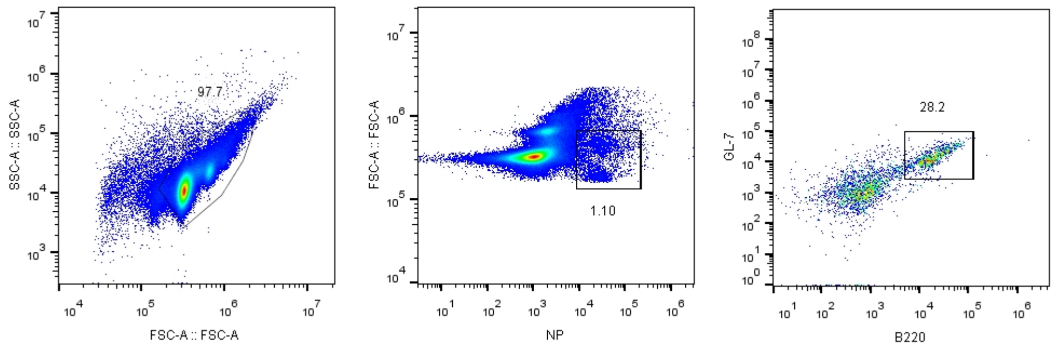

Fig. 2E

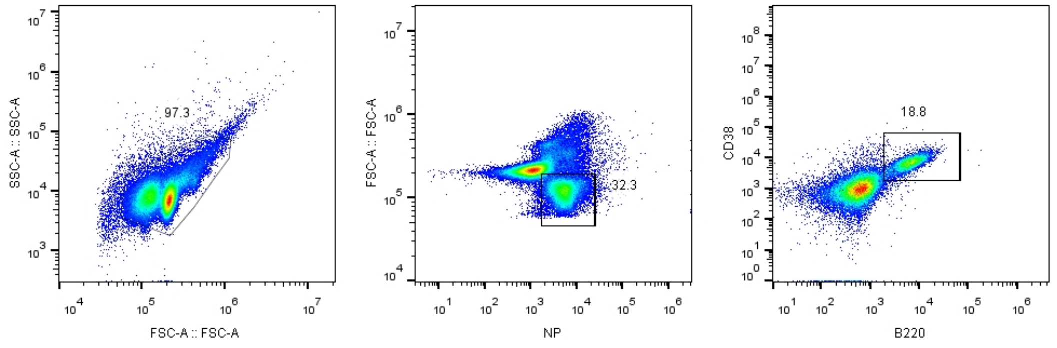

Fig. 2F

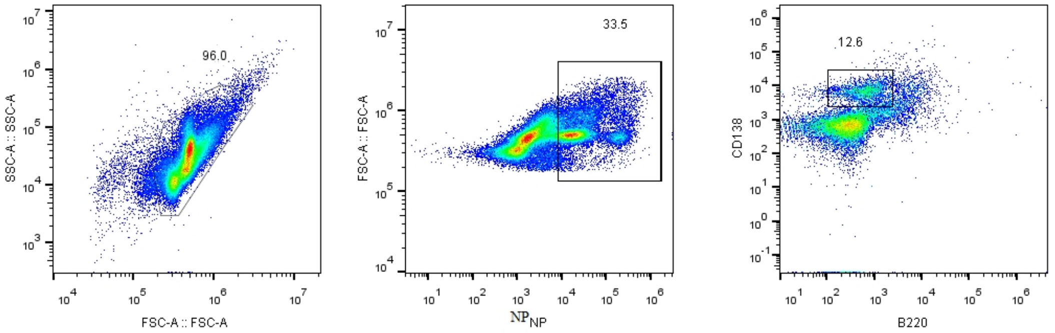
96.0
SSC-A :: SSC-A
FSC-A :: FSC-A
33.5
FSC-A :: FSC-A
NP NP
12.6
CD138
B220
